# Extracellular vesicle markers in relation to obesity and metabolic complications in patients with manifest cardiovascular disease

**DOI:** 10.1186/1475-2840-13-37

**Published:** 2014-02-05

**Authors:** Mariëtte EG Kranendonk, Dominique PV de Kleijn, Eric Kalkhoven, Danny A Kanhai, Cuno SPM Uiterwaal, Yolanda van der Graaf, Gerard Pasterkamp, Frank LJ Visseren

**Affiliations:** 1 Department of Vascular Medicine, University Medical Centre Utrecht (UMCU), F02.126, Heidelberglaan 100, Utrecht 3584 CX, The Netherlands; 2 Department of Metabolic Diseases, UMCU, Utrecht, The Netherlands; 3 Experimental Cardiology Laboratory, UMCU, Utrecht, The Netherlands; 4 Cardiovascular Research Institute & Surgery, NUHS, 5 Lower Kent Ridge Road, Singapore 119074, Singapore; 5 Julius Centre for Health Sciences and Primary Care, UMCU, Utrecht, The Netherlands

**Keywords:** Extracellular vesicle markers, Obesity, hsCRP, Metabolic syndrome, Type 2 diabetes

## Abstract

**Background:**

Alterations in extracellular vesicles (EVs), including exosomes and microparticles, contribute to cardiovascular disease. We hypothesized that obesity could favour enhanced release of EVs from adipose tissue, and thereby contribute to cardiovascular risk via obesity-induced metabolic complications. The objectives of this study were: 1) to investigate the relation between the quantity, distribution and (dys) function of adipose tissue and plasma concentrations of atherothrombotic EV-markers; 2) to determine the relation between these EV-markers and the prevalence of the metabolic syndrome; and 3) to assess the contribution of EV markers to the risk of incident type 2 diabetes.

**Methods:**

In 1012 patients with clinically manifest vascular disease, subcutaneous and visceral fat thickness was measured ultrasonographically. Plasma EVs were isolated and levels of cystatin C, serpin G1, serpin F2 and CD14 were measured, as well as fasting metabolic parameters, hsCRP and adiponectin. The association between adiposity, EV-markers, and metabolic syndrome was tested by multivariable linear and logistic regression analyses. As sex influences body fat distribution, sex-stratified analyses between adipose tissue distribution and EV-markers were performed. The relation between EV-markers and type 2 diabetes was assessed with Cox regression analyses.

**Results:**

Higher levels of hsCRP (*β* 5.59; 95% CI 3.00–8.18) and lower HDL-cholesterol levels (*β-*11.26; 95% CI −18.39 – -4.13) were related to increased EV-cystatin C levels, and EV-cystatin C levels were associated with a 57% higher odds of having the metabolic syndrome (OR 1.57; 95% CI 1.19–2.27). HDL-cholesterol levels were positively related to EV-CD14 levels (*β* 5.04; 95% CI 0.07–10.0), and EV-CD14 levels were associated with a relative risk reduction of 16% for development of type 2 diabetes (HR 0.84, 95% CI 0.75–0.94), during a median follow up of 6.5 years in which 42 patients developed type 2 diabetes.

**Conclusions:**

In patients with clinically manifest vascular disease, EV-cystatin C levels were positively related, and EV-CD14 levels were negatively related to metabolic complications of obesity.

## Background

Obese individuals are at increased risk of developing cardiovascular disease (CVD), a consequence of adipose tissue (AT) expansion and subsequent dysfunction [1,2]. In particular the expansion of visceral AT (VAT), rather than subcutaneous AT (SAT), is an independent risk factor for cardiovascular morbidity and mortality [3,4]. VAT expansion results in local inflammation, characterized by hypertrophic adipocytes and increased influx of pro-inflammatory macrophages and cytotoxic T cells, contributing to elevated plasma levels of interleukin-6 (IL-6) and high sensitive C-reactive protein (hsCRP) [5-7]. Simultaneously, expression of the anti-inflammatory adipokine adiponectin is downregulated in adipocytes [8]. This inflammatory milieu leads to metabolic complications that predispose to the development of CVD, including low-grade systemic inflammation, insulin resistance [9] and development of the metabolic syndrome and type 2 diabetes [10,11]. Further emphasizing the link between VAT and CVD are sex differences in body fat distribution, where males are both prone to develop abdominal obesity due to accumulation of visceral fat and suffer from a higher incidence of metabolic and cardiovascular disease [12].

Nevertheless, the pathophysiological mechanisms underlying the development of obesity-induced CVD are still poorly understood, and a biomarker indicating the obese individuals at risk would be extremely useful [13]. Extracellular vesicles (EVs) or EV-associated molecules are promising biomarkers in a variety of pathological settings [14,15]. EVs include microvesicles, microparticles and exosomes which are nanometre sized membrane vesicles secreted by all eukaryotic cells [16]. These vesicles reflect the state of a cell, and serve as messenger vehicles containing cell-specific cytosolic and membrane-bound proteins and RNA. As such, EVs can modify and activate target cells in a paracrine or endocrine fashion [17]. Important biological functions of EVs have been reported in a variety of (patho) physiological processes, including immune responses, inflammation, tumorigenesis and endothelial dysfunction [14].

Our group has previously shown that four EV-associated proteins cystatin C, serpin G1, serpin F2 and CD14 are associated with atherosclerotic plaque formation in patients with clinically manifest vascular disease, and that three of these (cystatin C, serpin F2 and CD14) related to an increased risk for cardiovascular morbidity and mortality in these patients [18]. Increased levels of cystatin C, a cysteine protease, are observed in obese subjects and associate with CVD [19,20]. Serpin G1, better known as C1 inhibitor, is an inhibitor of kallikrein and factor IIa [21]. Serpin F2 or α-2-antiplasmin, is the primary inhibitor of plasmin in the circulation [22]. CD14, a monocyte marker, is present on monocyte-derived EVs and such EVs have been shown to be capable of stimulating endothelial cells [23]. Interestingly, there appears to be considerable overlap between the inflammatory processes underlying AT inflammation and those that underlie atherosclerosis, since inflammatory cytokines such as hsCRP and IL-6, as well as immune cells such as macrophages, play crucial roles in the onset and progression of both conditions [24,25]. Furthermore, AT inflammation contributes to a pro-thrombotic state by secretion of adipokines such as TNF-α, leptin and IL-1β, which triggers blood coagulation by inducing the synthesis of tissue factor [26,27]. Therefore, it can be hypothesized that adiposity contributes to an elevated risk of developing CVD via increased production of EVs or EV-related molecules such as EV-associated cystatin C, serpin G1, serpin F2 and CD14.

While we previously reported on possible determinants for protein levels of these four EV-markers [18], we now aimed to further investigate the potential etiologic relation of AT quantity, AT distribution and metabolic parameters of AT (dys) function with EV-associated plasma protein levels of cystatin C, serpin G1, serpin F2 and CD14. Furthermore, the relation of these EV-markers to metabolic syndrome and incident type 2 diabetes in patients with clinically manifest vascular disease was investigated.

## Methods

### Study design and patient population

The study cohort consists of patients participating in the Second Manifestations of ARTerial disease (SMART) study, an ongoing prospective single-centre cohort study in patients with manifest arterial disease or cardiovascular risk factors at the University Medical Centre Utrecht (UMCU) which started in September 1996 [28]. The study was approved by the Ethics Committee of the UMCU and all patients gave their written informed consent. All patients included in the SMART-study were asked to complete a health questionnaire covering medical history, risk factors, smoking habits and medical treatment. A standardized diagnostic protocol was followed consisting of physical examination and laboratory testing in a fasting state. A detailed study rationale and description are published elsewhere [28].

For the present study, a total of 1062 patients with clinically manifest vascular disease at study inclusion, who enrolled in the SMART cohort between May 2001 until December 2005 were included. In this subset of patients, concentrations of extracellular vesicle (EV)-associated cystatin C, serpin G1, serpin F2 and CD14 were determined, as described previously [18]. Of the eligible 1062 patients, 2 had insufficient material for measurements of the four EV-markers, and 48 patients with hsCRP levels above 15 mg/L were excluded, leaving a final number of 1012 patients eligible for cross-sectional analysis. To reduce bias and improve statistical efficiency, missing values for smoking status (n = 6), estimated glomerular filtration rate ((eGFR), based on the modification of diet in renal disease (MDRD) formula) (n = 12), body mass index (n = 1), waist circumference (n = 42), visceral fat thickness (n = 8) and subcutaneous fat thickness (n = 19) were completed in the dataset by single imputation. There were no substantial differences in outcomes when we compared the results with complete case analysis (i.e. exclusion of cases with missing values). For analyses with HOMA-IR included in the model, only subjects included in the cohort after August 2003 were considered (n = 530), because plasma insulin levels were measured since that date.

### Extracellular vesicle marker measurements

EVs were isolated using Exoquick™ (SBI) according to the manufacturer’s protocol described previously [18]. Briefly, 150 μl EDTA plasma was centrifuged for 15 min at 3000×*g.* The supernatant was filtered over a 0.45 μm Spin-X filter (Corning), which was flushed with preheated PBS (37°C) and 38 μl ExoQuick™ solution was added to the filtrate. After vortexing, the sample was stored overnight at 4°C. The following day, the sample was centrifuged at 1500 × *g* for 30 min at room temperature, and the pelletwas lysed in 100 μl Roche Complete Lysis‒M with protease inhibitors (EDTA free). The sample was filtered over a 0.22 μm Spin-X filter (Corning) and protein concentration was determined using a Pierce® BCA Protein Assay Kit (Pierce Biotechnology, Rockford, USA), in order to correct the amount of measured EV-marker for the total amount of protein present in the EVs. Samples were stored at −80°C. After thawing, the lysed sample was diluted 20x with Roche complete Lysis-M buffer, and 50 μl was analysed in a multiplex immunoassay on levels of cystatin C, serpin G1, serpin F2 and CD14 using a Biorad Bioplex 200 system as described before [29]. Capture antibody, biotinylated detection antibody and antigen of all 4 proteins were purchased from R& D systems. A full description of preceding biomarkers proteomics discovery work is provided previously [18].

### Measurements of AT quantity

Visceral adipose tissue (VAT) and subcutaneous adipose tissue (SAT) thickness were quantified by ultrasonographic intra-abdominal fat measurement, performed by well-trained registered vascular technologists in a certified vascular laboratory. Ultrasonographic measurements were made in supine position using an ATL HDI 3000 (Philips Medical Systems, Eindhoven, The Netherlands) with a C 4–2 transducer [30]. An inter-observer coefficient of variation of 5.4% was found for ultrasound measurements of intra-abdominal fat, indicating good reproducibility [30]. Waist circumference (WC) was measured as the circumference in centimetres halfway between the lower rib and the iliac crest. Body Mass Index (BMI), the weight in kilograms divide by the square if the height in meters, was computed after a standardized anthropometric measurement protocol.

### Measurements of metabolic parameters of adipose tissue (dys) function

Serum concentration of adiponectin was measured by Luminex immunoassay (Biorad, Munich, Germany) as described previously [31]. Plasma insulin was measured with an immunometric technique on an IMMULITE 1000 Analyzer (Diagnostic Products Corporation, Los Angeles, USA). Insulin measurements below the lower limit of detection of 2 mIU/L (n = 1) were left out of the analysis. The value for insulin resistance was assessed by the formula: homeostasis model assessment parameter of insulin resistance (HOMA-IR) = fasting serum glucose (mmol/L) × fasting serum insulin (mIU/L))/22.5 [32], and was only performed in patients without antihyperglycaemic drugs. High-density lipoprotein cholesterol (HDL-C) in plasma was determined using a commercial enzymatic kit (Boehringer-Mannheim) after precipitation of low density lipoprotein cholesterol (LDL-C) and very low density lipoprotein cholesterol (VLDL-C) with sodiumphosphotungstatemagnesium chloride. hsCRP levels were determined by immunonephelometry (Nephelometer Analyser BN II, Dade-Behring, Marburg, Germany), with a lower detection limit of the test of 0.2 mg/L. As high hsCRP levels may have a different pathophysiological origin than low-grade inflammation as seen in obesity and vascular diseases, subjects with hsCRP levels > 15 mg/L were excluded from analysis.

### Follow up

To assess the incidence of diabetes, all patients that had been included until December 2005 without diabetes at baseline received a questionnaire in the period between June and December 2006 to assess the incidence of type 2 diabetes after study inclusion. After 2006, all patients were biannually asked to complete this questionnaire. Patients were asked whether they had diabetes and if ‘yes’, they received a supplementary questionnaire regarding date of diagnosis, initial and current treatment (oral medication or insulin). All diabetes cases were audited and classified by two independent physicians. Cross-validation with the hospital diagnosis registry revealed that none of the patients who reported not to have diabetes had a physician’s diagnosis of diabetes. Duration of follow-up was defined as the period between the date of study inclusion and the date of incident type 2 diabetes, date of loss to follow-up or the preselected closing date of 1 March 2010. From 1996 until 1 March 2010, 35 of 937 patients (3.5%) were lost to follow-up.

### Data analyses

Baseline characteristics were reported in Table 1, of which data regarding body fat are shown stratified for sex due to known sex differences in body fat distribution. Continuous variables are expressed as mean ± standard deviation (SD) when normal distributed or as median (interquartile range) in case of skewed distribution. Categorical variables are expressed as numbers (percentage). Variables with skewed distributions (cystatin C, serpin G1, serpin F2, CD14, HOMA-IR, adiponectin and hsCRP) were transformed to fulfil linear regression criteria.

**Table 1:** Baseline characteristics

		**N = 1012**	**P value**
Age (years)		59 ± 10	
Male sex, n (%)		804 (79)	
Obesity (BMI >30 (kg/m^2^); n (%))		178 (18)	
BMI (kg/m^2^)	Males (n = 804)	26.8 ± 3.6	0.479
	Females (n = 208)	27.1 ± 4.7	
Waist circumference (cm)			< 0.001
	Males (n = 804)	97 ± 10	
	Females (n = 208)	88 ± 12	
Intra-abdominal fat (cm)			< 0.001
	Males (n = 804)	9.9 ± 2.6	
	Females (n = 208)	8.3 ± 2.4	
Subcutaneous fat (cm)			< 0.001
	Males (n = 804)	2.3 ± 1.3	
	Females (n = 208)	3.0 ± 1.5	
Type 2 diabetes, n (%)		158 (15)	
Metabolic syndrome, n (%)^†^		517 (51)	
Blood pressure (mmHg)			
	Systolic	144 ± 22	
	Diastolic	83 ± 12	
Smoking, n (%)			
	Never	171 (17)	
	Ever	464 (46)	
	Current	377 (37)	
Pack years smoking		20.8 (6.4 – 35.0)	
**Metabolic parameters**			
Cholesterol (mmol/L)		4.9 ± 1.0	
LDL-cholesterol (mmol/L)		2.8 ± 0.9	
HDL-cholesterol (mmol/L)		1.3 (1.0 – 1.5)	
Triglycerides (mmol/L)		1.5 (1.1 – 2.1)	
Glucose (mmol/L)		6.3 ± 1.8	
Insulin (mU/L)		10.0 (7.0 – 14.0)	
HOMA-IR		2.5 (1.6 – 3.9)	
hsCRP (mg/L)		1.9 (0.9 – 3.8)	
Adiponectin (μg/ml)		19.7 (12.5 – 33.2)	
eGFR (ml/min/1.73 m^2^)^‡^		77 ± 18	
Creatinine (mmol/l)		93 ± 44	
**History of vascular disease, n (%)**			
Cerebrovascular disease		265 (26)	
Coronary artery disease		597 (59)	
Peripheral artery disease		242 (24)	
Aneurysm of the abdominal aorta		99 (10)	
**Medication use, n (%)**			
Platelet-aggregation inhibitors		766 (76)	
Blood pressure-lowering agents		734 (73)	
Lipid-lowering agents		685 (68)	
Oral anticoagulants		78 (8)	
Anti-hyperglycaemic agents		96 (10)	

Values are expressed in n (%), mean ± SD or median (interquartile range). ^†^Defined according to the National Cholesterol Education Program ATPIII-revised guidelines. ^‡^eGFR, estimated glomerular filtration rate, estimated by the modification of diet in renal disease equation (MDRD). BMI, body mass index; LDL, low-density lipoprotein; HDL, high-density lipoprotein; HOMA-IR, homeostasis model assessment parameter of insulin resistance; hsCRP, high sensitive C-reactive protein.

Differences in baseline concentrations of EV-markers in different metabolic groups were calculated by analysis of covariance (ANCOVA), corrected for age, sex and eGFR. Data in Table 2 are displayed in the original scale of measurement. However, to fulfil ANCOVA’s assumptions a logarithmic transformation was applied to EV-cystatin C, EV-serpin G1 and EV-serpin F2 data, and a square root transformation to EV-CD14 data prior to formal analysis [33]. No interaction between covariates and independent variables was observed. For several metabolic groups (waist circumference, visceral adipose tissue or subcutaneous adipose tissue), different distribution or cut off values are known for males and females. Therefore, male and female patients were divided separately into two groups based on the median value or appropriate cut off value for that sex and then combined into sex-pooled groups.

**Table 2:** Baseline concentrations of EV-markers (pg/μg) in metabolically (un) compromised groups

	**EV-cystatin C**	**EV-serpin G1**	**EV-serpin F2**	**EV-CD14**
**Obesity**				
BMI < 30 kg/m^2^ (n = 829)	9.5 (7.4 – 12.1)	122.7 (86.0 – 167.1)	36.8 (21.9 – 56.7)	11.56(9.6 – 14.0)
BMI > 30 kg/m^2^ (n = 178)	9.1 (7.1 – 12.1)	121.5 (83.4 – 176.1)	38.1 (21.4 – 61.2)	11.1 (9.3 – 13.4)
*p value*	0.776	0.081	0.831	0.139
Visceral obesity*				
No (n = 645)	9.1 (7.2 – 11.5)	120.5 (82.5 – 165.7)	36.2 (21.9 – 56.1)	11.4 (9.3 – 13.9)
Yes (n = 364)	10.0 (7.5 – 13.1)	126.4 (89.3 – 173.7)	38.6 (22.1 – 61.1)	11.8 (9.7 – 13.9)
*p value*	0.614	0.503	0.224	0.066
**Visceral adipose tissue †**				
VAT < median (n = 494)	9.1 (7.2 – 11.7)	121.4 (85.9 – 166.1)	38.7 (23.2 – 57.9)	11.7 (9.7 – 14.2)
VAT > median (n = 515)	9.7 (7.4 – 12.8)	123.8 (85.1 – 172.9)	35.6 (19.7 – 57.4)	11.4 (9.4 – 13.7)
*p value*	0.540	0.765	0.129	0.257
**Subcutaneous adipose tissue ‡**				
SAT < median (n = 484)	9.8 (7.6 – 12.6)	124.3 (87.6 – 170.6)	37.6 (22.5 – 56.8)	11.8 (9.6 – 14.5)
SAT > median (n = 525)	9.1 (7.1 – 11.7)	121.0 (84.0 – 167.8)	36.8 (21.4 – 58.3)	11.3 (9.5 – 13.4)
*p value*	0.744	0.335	**0.002**	0.512
Hyperlipidaemia**				
No (565)	9.4 (7.3 – 12.1)	120.9 (87.4 – 166.8)	35.4 (20.7 – 56.7)	10.9 (9.1 – 13.1)
Yes (417)	9.4 (7.2 – 12.1)	124.2 (82.5 – 174.4)	40.2 (23.8 – 60.9)	12.4 (10.3 – 14.8)
*p value*	0.981	0.344	**0.013**	**< 0.001**
**Metabolic syndrome**				
No (n = 492)	9.0 (7.1 – 11.4)	121.0 (81.7 – 167.2)	36.5 (21.9 – 56.6)	11.5 (9.4 – 13.9)
Yes (n = 517)	9.8 (9.1 – 12.8)	124.1 (88.99 – 170.4)	37.6 (22.0 – 58.7)	11.6 (9.6 – 13.8)
*p value*	**0.010**	0.450	0.572	0.499
**Baseline type 2 diabetes**				
No (n = 850)	9.3 (7.3 – 11.9)	124.9 (87.9 – 172.9)	37.1 (21.9 – 57.4)	11.5 (9.4 – 13.9)
Yes (n = 158)	10.1 (7.4 – 13.2)	111.0 (78.4 – 158.5)	36.9 (22.2 – 59.8)	12.0 (9.9 – 13.8)
*p value*	0.050	**0.010**	0.909	0.111
**Development of type 2 diabetes**				
No (n = 745)	9.0 (7.1 – 11.5)	122.3 (86.9 – 168.4)	36.0 (21.6 – 55.7)	11.3 (9.3 – 13.5)
Yes (n = 86)	9.5 (7.3 – 12.7)	122.9 (88.7 – 163.9)	34.7 (20.9 – 55.6)	11.0 (11.1 – 13.8)
*p value*	0.768	0.768	0.889	**< 0.001**

P value is calculated by analysis of covariance, corrected for age, sex and eGFR. P values are calculated on log or square root transformed EV-marker values, but for ease of interpretation, the results of calculations are transformed back to original baseline levels of EV markers in pg/μg. *Visceral obesity is defined as waist circumference above 88 cm for females, or above 102 cm for males. **Hyperlipidaemia is defined as total cholesterol > 5.0 mmol/L, LDL-cholesterol > 3.2 mmol/L and/or use of lipid lowering agents. **†**Visceral adipose tissue values are sex pooled. For males, mean = 9.9 cm, for females mean = 8.3 cm.**‡** Subcutaneous adipose tissue values are sex pooled. For males, mean = 9.9 cm, for females mean = 8.3 cm.

### Adipose tissue quantity and EV-markers

As body fat distribution differs between males and females, and is differently related to CVD [34], potential effect modification on the relationship between adiposity and EV markers was investigated by entering an interaction term for sex to the most complete adjusted model. Effect modification of sex could be demonstrated in the relation between SAT and BMI with serpin F2 (p-values for interaction were 0.023 and 0.014 respectively), and between WC and CD14 (p-value for interaction was 0.016). Therefore, separate analyses were performed for males and females. Multivariable linear regression analysis was used to evaluate relations between parameters of AT quantity (VAT, SAT, WC and BMI) and EV-markers (EV-associated protein levels of cystatin C, serpin G1, serpin F2 and CD14), expressed as beta (*β*) regression coefficients and 95% confidence intervals (95% CI). Analyses were adjusted for age, current smoking, eGFR, type 2 diabetes, blood pressure lowering medication, lipid lowering medication and year of inclusion in SMART. As subjects were included over a time period of 6 years, baseline measurements might be slightly different, and adjustment for year of inclusion was added to the final model.

### Metabolic parameters of adipose tissue (dys) function and EV-markers

The relation between metabolic parameters of AT (dys) function (plasma levels of adiponectin, hsCRP and HDL-cholesterol and HOMA-IR) and EV-markers was assessed by multivariable linear regression analysis adjusted for age, sex, current smoking, eGFR, type 2 diabetes, blood pressure lowering medication, lipid lowering medication, platelet aggregation inhibitors and year of inclusion in SMART. No interaction for sex could be demonstrated in the relation between metabolic parameters and EV-markers. For presentation purposes, log or square root transformed EV marker values were multiplied by 100. We did not correct for multiple testing, as all analysis performed were strictly hypothesis driven.

### EV-markers and metabolic syndrome or incident type 2 diabetes

Multivariable logistic regression analyses were used to assess the relation between the EV markers and the metabolic syndrome. The Adult Treatment Panel (ATP) III criteria were taken for the definition of the metabolic syndrome [10]. No interaction for sex could be demonstrated in the relation between the metabolic syndrome and the EV-markers. Results are expressed as odds ratios (OR) with corresponding 95% CI. Analyses were adjusted for age, sex, current smoking, eGFR and year of inclusion in SMART.

The relation between EV-markers and incident type 2 diabetes was quantified with Cox proportional hazards analysis, and results are expressed as hazard ratios (HR) with corresponding 95%CI. Analyses were adjusted for age, sex, current smoking, eGFR, year of inclusion in SMART, metabolic syndrome and HOMA-IR. The proportional hazards assumptions were formally tested with the Schoenfeld test. No significant non-proportionality (p < 0.05) was observed. No interaction for sex could be demonstrated in the relation between these metabolic parameters and the EV-markers.

Analyses were performed in SPSS version 20 (SPSS, Chicago, Illinois, USA) and R version 2.15.2.

## Results

### Baseline characteristics

The patient characteristics are summarized in Table 1. The average age was 59 ± 10 years and 79% were males. In total 18% of the patients was obese. Mean VAT thickness and WC were higher in males than females (VAT males: 9.9 ± 2.6 cm, females: 8.3 ± 2.4 cm, WC males: 97 ± 10 cm, females: 88 ± 12 cm), and mean SAT thickness was higher in females than in males (males: 2.3 ± 1.3 cm, females: 3.0 ± 1.5 cm). 51% of the patients had metabolic syndrome, defined by the Adult Treatment Panel (ATP) III criteria [10], of which 36% had central obesity, 94% were hypertensive, 39% had hypertriglyceridemia, 29% had low HDL-cholesterol levels and 63% had an impaired fasting glucose.

In Table 2, baseline levels of circulating EV-cystatin C, EV-serpin G1, EV-serpin F2 and EV-CD-14 are presented among different metabolically (un) comprised groups. EV-cystatin C levels were higher in patients with metabolic syndrome compared to patients without metabolic syndrome (9.84 pg/μg versus 9.97 pg/μg, p = 0.010). EV-serpin G1 levels were lower in patients with baseline type 2 diabetes compared to patients without type 2 diabetes at baseline (111.0 pg/μg versus 124.9 pg/μg, p = 0.010). EV-serpin F2 levels were lower in patients with more subcutaneous fat (36.81 pg/μg versus 37.37 pg/μg, p = 0.002) and higher in patients suffering from hyperlipidaemia compared to patients without hyperlipidaemia (40.16 pg/μg versus 35.44 pg/μg, p = 0.013). EV-CD14 levels were higher in patients suffering from hyperlipidaemia (12.43 pg/μg versus 10.90 pg/μg, p < 0.001) and lower in patients who developed incident type 2 diabetes compared to patients who did not develop incident type 2 diabetes (11.04 pg/μg versus 11.26 pg/μg, p < 0.001).

### Relation between adipose tissue quantity and EV-markers

**Figure 1:**
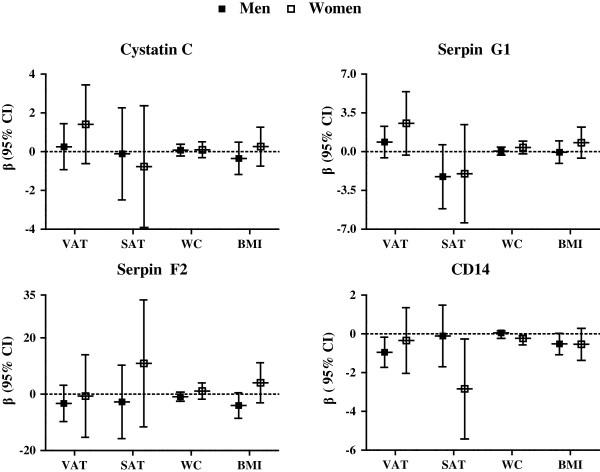
**Relation between visceral fat, subcutaneous fat, waist circumference and BMI and EV-markers in males and females with manifest cardiovascular disease.** Beta regression coefficients (*β*) with 95% confidence interval (CI) indicates the difference in log EV-cystatin C, log EV-serpin G1, square root EV-serpin F2 or log EV-CD14 levels per unit increase in AT parameter, adjusted for age, current smoking, eGFR, type 2 diabetes, blood pressure lowering medication, lipid lowering medication and year of inclusion in SMART.

In Figure 1, the results of four different measures of adiposity in relation to EV-markers are presented. None of the measures of adiposity were significantly related to increased EV-levels in the fully adjusted multivariable regression model. In contrast, fat thickness was related to decreased plasma EV-CD14 levels in both males and females. Interestingly, in males an increase in VAT thickness was significantly related to lower EV-CD14 levels (*β* -0.95; 95% CI −1.73 – -0.17) while in females SAT thickness was significantly related to lower EV-CD14 levels (*β* -2.8; 95% CI −5.42 – -0.26, Figure 1). Additional file 1: Table S1 includes linear regression coefficients with 95% CI of unadjusted, partially adjusted and fully adjusted multivariable models for the relation between measures of obesity and EV-markers.

### Relation between metabolic parameters of adipose tissue (dys) function and EV-markers

**Figure 2:**
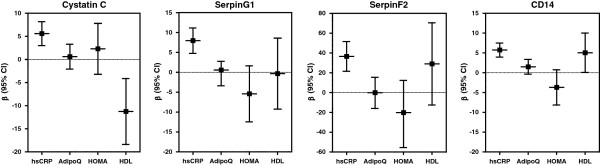
**Relation between metabolic parameters of adipose tissue (dys) function and EV-markers in patients with manifest cardiovascular disease.***β* with 95% CI indicates the difference in log EV-cystatin C, log EV-serpin G1, square root EV-serpin F2 or log EV-CD14 levels per unit increase in log hsCRP, log adiponectin, log HOMA-IR or HDL-cholesterol, adjusted for age, sex, current smoking, eGFR, type 2 diabetes, blood pressure lowering medication, lipid lowering medication, platelet aggregation inhibitors and year of inclusion in SMART. AdipoQ: adiponectin.

Linear regression coefficients with 95% CI of relations between metabolic parameters of AT (dys) function and EV-markers are shown in Figure 2 (fully adjusted model) and Additional file 1: Table S2 (including models with and without adjustment for confounding variables). Higher hsCRP levels were strongly related to higher EV-marker levels (cystatin C *β* 5.59; 95% CI 3.00–8.18, serpin G1 *β* 7.96; 95% CI 4.76–11.16, serpin F2 *β* 36.52; 95% CI 21.49–51.55, CD14 *β* 5.72; 95% CI 3.94–7.49; Figure 2, Additional file 1: Table S2). Additional adjustment for dyslipidaemia, history of vascular disease, blood pressure and the metabolic syndrome did not affect the determinant-outcome relation (data not shown).

Plasma adiponectin concentration and HOMA-IR levels were not significantly related to any of the EV-markers.

Low HDL-cholesterol was significantly related to higher EV-cystatin C levels (*β* -11.26; 95% CI −18.39 – -4.13), while high HDL cholesterol was significantly related to higher EV-CD14 levels *(β* 5.04; 95% CI 0.07 – 10.00; Figure 2, Additional file 1: Table S2).

### EV-markers in relation to the metabolic syndrome

**Figure 3:**
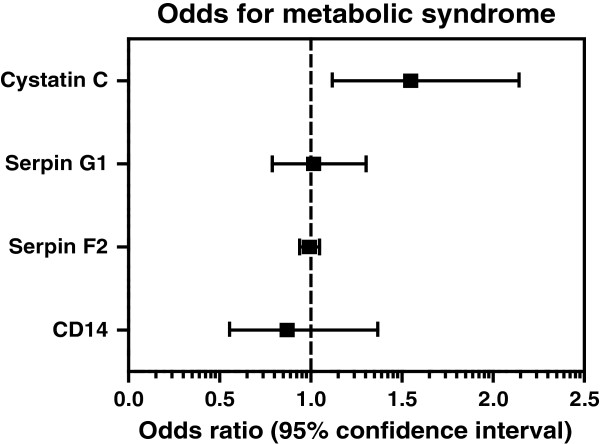
**Relation between EV-markers and metabolic syndrome in patients with manifest cardiovascular disease.** Odds ratios with 95% CI indicate the odds for metabolic syndrome per increase in log EV-cystatin C, log EV-serpin G1, square root EV-serpin F2 or log EV-CD14 concentration, adjusted for age, sex, current smoking, eGFR and year of inclusion in SMART.

Higher EV-cystatin C levels were significantly related to a 57% higher prevalence of the metabolic syndrome (OR 1.57, 95% CI 1.19–2.27) while no relation was observed between the other EV markers and prevalence of the metabolic syndrome (Figure 3). Unadjusted and partially adjusted multivariable models are provided in Additional file 1: Table S3.

### EV-markers in relation to incident type 2 diabetes

**Figure 4:**
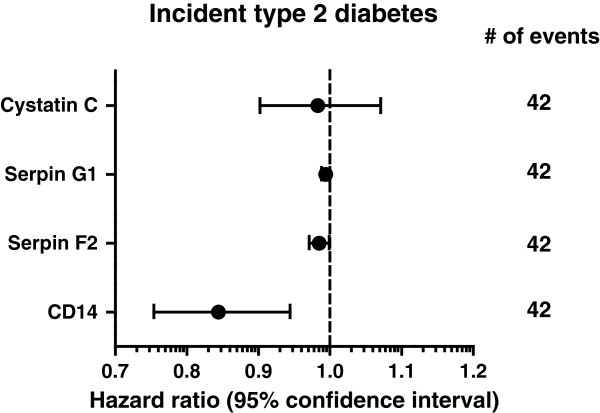
**EV-markers and the risk of new onset type 2 diabetes in patients with manifest cardiovascular disease.** Hazard ratios with 95% CI indicate the relative risk for incident type 2 diabetes per increase in EV-cystatin C, EV-serpin G1, EV-serpin F2 or EV-CD14 concentration during 6.5 years (interquartile range 5.8–7.1 years) follow-up, adjusted for age, sex, current smoking, hsCRP, eGFR and year of inclusion in SMART.

During a median follow up of 6.5 years (interquartile range 5.8–7.1 years), 42 patients developed type 2 diabetes. As shown in Figure 4, higher levels of EV-markers were not related to an increased risk for type 2 diabetes. In fact, in patients with high EV-CD14 levels at baseline*,* a relative risk reduction of 16% for development of type 2 diabetes was observed (HR 0.84, 95% CI 0.75–0.94; Figure 4). Unadjusted and partially adjusted multivariable models are provided in Additional file 1: Table S4.

## Discussion

In the present study, we assessed the relation between AT quantity, AT distribution and metabolic parameters of AT (dys) function and plasma levels of four CVD-associated EV-markers. Furthermore, the relation between these EV-markers and metabolic syndrome or incident type 2 diabetes in patients with clinically manifest vascular disease was investigated. We show that EV-cystatin C was positively related to metabolic complications of obesity, including low-grade systemic inflammation, low HDL-cholesterol levels and metabolic syndrome. In contrast, EV-CD14 was inversely related to AT abundance and dyslipidaemia, and was moreover related to a relative risk reduction for the development of type 2 diabetes.

Cystatin C has previously been shown to be elevated in obese subjects [35], to be secreted by AT *in vitro*[36] and to be associated with the metabolic syndrome [37]. Furthermore, cystatin C has been associated with prediabetes and cardiovascular disease, independent of renal function [18,38,39]. In contrast to other studies performed in patients without known cardiovascular disease, we could not demonstrate a relation between obesity and circulating EV-cystatin C-levels, nor were HOMA-IR levels significantly related to EV-cystatin C levels. As we have specifically studied a cohort of patients with clinically manifest vascular disease, a difference in cohort characteristics between these studies may account for discordant results. Nonetheless, we did find strong relations between alternative parameters of adipose tissue dysfunction with EV-cystatin C levels, such as low-grade systemic inflammation and low HDL levels. These findings may suggest that AT dysfunction rather than AT abundance is a more important determinant of EV-cystatin C levels, at least in patients with cardiovascular disease. Furthermore, in concordance with studies performed in healthy individuals [37], we observed a strong relation between EV-cystatin C and the metabolic syndrome in patients with clinically manifest vascular disease. Thus, the EV-marker cystatin C may be an important biomarker for CVD not only in healthy individuals but importantly also in patients with manifest vascular disease.

Since obesity is associated with a pro-thrombotic state, we hypothesized that obesity could contribute to circulating EV-serpin G1 and EV-serpin F2, both pro-coagulant markers. Serpin G1, better known as C1 inhibitor, is primarily involved in the inhibition of coagulation and atherosclerotic plaque formation [40], though its role in obesity has not been investigated. Serpin F2, or α-2-antiplasmin, is a major inhibitor of plasmin and thereby controls the coagulation system [22]. Previous studies reported a negative relation of VAT thickness and soluble plasma serpin F2 levels [41]. In our study, obesity was not related to EV-serpin F2 nor to EV-serpin G1, and both markers were not related to metabolic complications of obesity. However, EV-serpin G1 and EV-serpin F2 did show a strong positive relation with low-grade inflammation. These data suggest that in patients with manifest vascular disease, these markers might contribute to low grade inflammation, though both EV-serpin G1 and EV-serpin F2 appear to play no role in the pathophysiology of metabolic complications.

Surprisingly, we observed an inverse relation between EV-CD14 levels with obesity and obesity-related metabolic complications in patients with clinically manifest vascular disease. CD14 is expressed primarily by monocytes, which play important roles in obesity, obesity-induced AT inflammation and insulin resistance [5,42]. CD14 has furthermore been associated with the development of atherosclerosis and the recurrence of vascular events [18,43]. EVs secreted by monocytes express CD14, and these EVs are capable of inducing endothelial damage *in vitro*[23]. However, conflicting results have been reported by others, as soluble CD14 did not relate to endothelial damage in type 2 diabetic subjects [44], and a recent study showed that lower soluble CD14 levels were associated with an increase in BMI in both obese and non-obese patients [45]. Possibly, two different forms of CD14 studied (soluble CD14 versus membrane-bound CD14) may account for differences in observed relations between obesity and CD14. Although both soluble and membrane bound CD14 are involved in inflammatory signalling pathways, CD14 is only part of a receptor complex and soluble CD14 needs binding to a cellular signal-transducing receptor in order for cell activation to occur [46]. Furthermore, an excess of circulating soluble CD14 is believed to inhibit monocyte responses to inflammatory signals for membrane CD14 [42,46]. It is unclear whether EVs contain soluble or membrane bound CD14, and whether EV-associated CD14 is functional. Nonetheless, as EV-CD14 levels were associated with low grade inflammation mirrored by circulating hsCRP levels, it is tempting to speculate that high EV-CD14 levels might contribute to vascular risk via inflammatory pathways, but not via obesity-induced metabolic complications.

The mechanisms by which EV-markers could influence the development of type 2 diabetes remain elusive. EVs are regarded as tailor-made messengers for intercellular communication, as their unique composition allows transfer of signalling molecules to a wide variety of target tissues [17]. However, even though a negative relation was observed between EV-CD14 and incident type 2 diabetes in this study, this does not necessarily imply a direct role for CD14 in reduced progression of development of type 2 diabetes. Considering that CD14 is only part of a receptor complex, the functional role of either soluble or membrane CD14 in EVs remains elusive. It could be hypothesized that EVs containing high levels of CV14 are also enriched for insulin sensitizing molecules like adiponectin, as circulating adiponectin levels were positively related to circulating EV-CD14 levels in this study (Additional file 1: Table S2). Further studies are needed to evaluate the pathophysiological role of EV-CD14 in the development of metabolic complications of obesity.

As levels of EV-cystatin C and EV-CD14 can be partly explained by AT abundance and AT (dys) function, we questioned whether AT could actively secrete both EV-markers or whether AT (dysfunction) triggers other tissues for their release. Cell types potentially involved in the release of EV-cystatin C and EV-CD14 include activated monocytes, endothelial cells and platelets, which are all present in high numbers in atherosclerosis lesions [43,47]. However, evidence suggests that these cell types also play active roles in AT dysfunction, in which hypertrophic adipocytes induce endothelial stress and the recruitment of monocytes [5]. Furthermore, production of both CD14 and cystatin C is increased in AT of obese compared to lean subjects [36,42]. Therefore, AT itself could be capable of secreting these markers due to adipocyte hypertrophy, hypoxia and increased influx of pro-inflammatory cells such as macrophages. As AT is capable of secreting functional EVs as shown in mice and humans [48,49], it will be interesting to study whether these AT EVs contain the EV-markers assessed in the present study.

Strengths of this study include the large sample size of a well-characterized and relevant patient population, which allowed for adjustment of multiple relevant potential confounding factors. Furthermore, measurement of different fat compartments with ultrasound allowed for the assessment of the contribution of the different adipose tissues depots to the levels of circulating EV-markers, known to be associated with CVD. Limitations of this study include the fact that, due to the cross-sectional design, causality in the relationships remain unknown. Second, the study population consisted solely of patients with clinically manifest vascular disease, which may limit the generalization of the results to other cohorts.

## Conclusions

In patients with clinically manifest vascular disease, EV-cystatin C positively relates to metabolic complications of obesity, and may thus contribute to an increased cardiovascular risk through obesity associated metabolic dysfunction. In contrast, EV-CD14 levels were inversely related to visceral obesity in males and associated with a relative risk reduction for the development of type 2 diabetes.

## Abbreviations

AdipoQ: Adiponectin; AT: Adipose tissue; BMI: Body mass index; CVD: Cardio vascular disease; eGFR: Estimated glomerular filtration rate; EV: Extracellular vesicles; IL-6: Interleukin 6; HDL-C: High-density lipoprotein cholesterol; LDL-C: Low-density lipoprotein cholesterol; HOMA-IR: Homeostasis model assessment parameter of insulin resistance; hsCRP: High sensitive C-reactive protein; MDRD: Modification of diet in renal disease; SAT: Subcutaneous adipose tissue; SMART: Second manifestations of ARTerial disease; UMCU: University Medical Centre Utrecht; VAT: Visceral adipose tissue; VLDL: Very low-density lipoprotein cholesterol; WC: Waist circumference.

## Competing interest

The authors declare that they have no competing interests.

## Authors’ contributions

DPVK, GP and FLJV conceived and designed the research. MEGK, DAK, YG, DPVK, GP and FLJV performed the research. MEGK and DAK analysed the data. MEGK, EK and FLJV wrote the manuscript. EK, CSPM, GP, DAK, DPVK and YG contributed to discussion and final version of the manuscript. All authors read and approved the final manuscript.

## Supplementary Material

Additional file 1 **Regression coefficients with 95% CI of unadjusted, partially adjusted and fully adjusted multivariable models for the relation between measures of obesity and EV-markers, the relation between EV-markers with metabolic syndrome and incident type 2 diabetes. Table S1.** Relation between visceral fat, subcutaneous fat, waist circumference or BMI and EV-markers in males with clinically manifest arterial disease. **Table S2.** Relation between plasma concentrations of hsCRP and EV-markers in patients with clinically manifest arterial disease. **Table S3.** Relation between EV-markers and the metabolic syndrome in patients with clinically manifest arterial disease. **Table S4.** Relation between EV-markers and risk of onset type 2 diabetes in patients with clinically manifest arterial disease. Click here for file
