# Supplementary material for: Extracellular vesicle markers in relation to obesity and metabolic complications in patients with manifest cardiovascular disease
Source: Cardiovasc Diabetol. 2014 Feb 5;13:37. doi: 10.1186/1475-2840-13-37 (PMC3918107; doi:10.1186/1475-2840-13-37)
Supplement: Additional file 1 — Regression coefficients with 95% CI of unadjusted, partially adjusted and fully adjusted multivariable models for the relation between measures of obesity and EV-markers, the relation between EV-markers with metabolic syndrome and incident type 2 diabetes. Table S1. Relation between visceral fat, subcutaneous fat, waist circumference or BMI and EV-markers in males with clinically manifest arterial disease. Table S2. Relation between plasma concentrations of hsCRP and EV-markers in patients with clinically manifest arterial disease. Table S3. Relation between EV-markers and the metabolic syndrome in patients with clinically manifest arterial disease. Table S4. Relation between EV-markers and risk of onset type 2 diabetes in patients with clinically manifest arterial disease. [file 1475-2840-13-37-S1.docx]

**Supplementary data**

**Extracellular vesicle markers in relation to obesity and metabolic complications in patients with manifest cardiovascular disease.**

**EV-markers, obesity and metabolic complications**

Mariëtte E.G. Kranendonk, Dominique P.V. de Kleijn, Eric Kalkhoven, Danny A. Kanhai, Cuno S.P.M. Uiterwaal, Yolanda van der Graaf, Gerard Pasterkamp, Frank L.J. Visseren; on behalf of the SMART Study Group

**Corresponding author:** Prof. Frank L.J. Visseren, MD, PhD, Department of Vascular Medicine, University Medical Centre Utrecht, F02.126, Heidelberglaan 100, 3584 CX Utrecht, The Netherlands, Tel: +31-887557155, Fax: +31-302522693, Email: [f.l.j.visseren@umcutrecht.nl](mailto:f.l.j.visseren@umcutrecht.nl)

**Table S1a. Relation between visceral fat, subcutaneous fat, waist circumference or BMI and EV-markers in males with clinically manifest arterial disease.**

|  | Model | Log cystatin C (pg/µg)  N = 799  Mean = 2.22 | Log serpin G1(pg/µg)  N = 801 Mean = 4.82 | Sqrt serpin F2 (pg/µg)  N = 797  Mean = 5.98 | Log CD14 (pg/µg) N = 802 Mean = 2.43 |
| --- | --- | --- | --- | --- | --- |
|  |  | *β* (95%CI) | *β* (95%CI) | *β* (95%CI) | *β* (95%CI) |
| Visceral fat thickness Mean = 9.9 cm | I  II  III  IV | 1.00 (-0.30 – 2.31)  0.79 (-0.46 – 2.03) 0.41 (-0.77 – 1.58) 0.25 (-0.93 – 1.44) | 1.08 (-0.35 – 2.50)  1.05 (-0.37 – 2.46)  1.24 (-0.18 – 2.65)  0.87 (-0.56 – 2.29) | -3.43 (-9.85 – 2.99)  -3.74 (-10.07 – 2.59)  -3.69 (-10.09 – 2.70)  -3.28 (-9.71 – 3.15) | -0.78 (-1.61 – 0.05)  **-0.90 (-1.69 – -0.11)***  **-1.02 (-1.82– -0.23)***  **-0.95 (-1.73 – -0.17)*** |
| Subcutaneous fat thickness Mean = 2.3 cm | I  II  III  IV | -2.17 (-4.75 – 0.42) 0.10 (-2.42 – 2.62) 0.59 (-1.77 – 2.94)  -0.11 (-2.49 – 2.26) | -1.56 (-4.41 – 1.29) -1.77 (-4.66 – 1.11) -1.63 (-4.49 – 1.24)  -2.26 (-5.15 – 0.62) | -0.41 (-13.23 – 12.42)  0.61 (-12.34 – 13.55)  0.78 (-12.19 – 13.76)  -2.74 (-15.76 – 10.28) | -0.51 (-2.17 – 1.14)  0.43 (-1.19 – 2.05)  0.53 (-1.08 – 2.15)  -0.11 (-1.70 – 1.48) |
| Waist circumference  Mean = 97 cm | I  II  III  IV | 0.17 (-0.16 – 0.49) 0.10 (-0.22 – 0.41) 0.11 (-0.18 – 0.41)  0.08 (-0.22 – 0.38) | 0.10 (-0.26 – 0.45)  0.06 (-0.29 – 0.28)  0.15 (-0.21 – 0.50)  0.05 (-0.31 – 0.41) | -0.90 (-2.51 – 0.71)  -1.12 (-2.70 – 0.47)  -1.07 (-2.67 – 0.54)  -0.90 (-2.52 – 0.72) | 0.02 (-0.23 – 0.19)  -0.08 (-0.28 – 0.12)  -0.09 (-0.29 – 0.11)  0.05 (-0.24 – 0.15) |
| BMI  Mean = 26.8 kg/m^2^ | I  II  III  IV | -0.90 (-1.81 – 0.01)  -0.39 (-1.26 – 0.48) -0.40 (-1.23 – 0.42)  -0.35 (-1.18 – 0.49) | -0.14 (-1.14 – 0.86) -0.14 (-1.14 – 0.86) -0.09 (-0.91 – 1.09)  -0.05 (-1.06 – 0.97) | **-5.61 (-10.08 – -1.13)***  **-5.38 (-9.83 – -0.93)***  **-5.30 (-9.80 – -0.80)***  -4.04 (-8.58 – 0.51) | **-0.95 (-1.52 – -0.37)***  **-0.74 (-1.30 – -0.18)***  **-0.79 (-1.35 – -0.23)***  -0.52 (-1.08 – 0.03) |

Linear regression coefficients (*β)* with 95% confidence interval (CI) indicates the difference in log cystatin C, serpin G1, CD14 or square root EV serpin F2 concentration per unit increase in adipose tissue parameter. Estimated differences are based on linear regression models. Model I: univariable model; Model II: adjustment for age and current smoking; Model III: model II with additional adjustment for eGFR and type 2 diabetes; Model IV: model III with additional adjustment for blood pressure lowering medication, lipid lowering medication and year of inclusion. Bold values indicate significance, *p<0.05.

**Table S1b.**  **Relation between visceral fat, subcutaneous fat, waist circumference or BMI and EV-markers in females with clinically manifest arterial disease.**

|  | Model | Log cystatin C (pg/µg)  N = 207  Mean = 2.28 | Log serpin G1(pg/µg)  N = 206 Mean = 4.73 | Sqrt serpin F2 (pg/µg)  N = 206  Mean = 6.51 | Log CD14 (pg/µg) N = 207 Mean = 2.48 |
| --- | --- | --- | --- | --- | --- |
|  |  | *β* (95%CI) | *β* (95%CI) | *β* (95%CI) | *β* (95%CI) |
| Visceral fat thickness Mean = 8.3 cm | I  II  III  IV | **3.20 (0.69 – 5.71)*** 2.06 (-0.32 – 4.43) 1.51 (-0.65 – 3.68) 1.41 (-0.62 – 3.44) | 2.47 (-0.24 – 5.19)  2.38 (-0.34 – 5.10)  2.59 (-0.25 – 5.43)  2.56 (-0.31 – 5.42) | -0.37 (-14.91 – 14.17)  3.17 (-11.19 – 17.54)  -0.30 (-15.35 – 14.75)  -0.66 (-15.31 – 14.00) | 0.40 (-1.29 – 2.09)  -0.12 (-1.78 – 1.55)  -0.35 (-2.09 – 1.39)  -0.34 (-2.04 – 1.35) |
| Subcutaneous fat thickness Mean = 3.1 cm | I  II  III  IV | -1.12 (-5.09 – 2.85) 0.50 (-3.22 – 4.22) 1.44 (-1.79 – 4.68)  -0.77 (-3.91 – 2.37) | -2.34 (-6.56 – 1.88) -1.20 (-6.22 – 2.23) -1.76 (-6.01 – 2.49)  -1.99 (-6.42 – 2.45) | **25.28 (2.91 – 47.66)***  **22.62 (0.54 – 44.69)***  20.90 (-1.29 – 43.09)  10.91 (-11.59 – 33.41) | -2.47 (-5.09 – 0.14)  -1.76 (-4.34 – 0.81)  -1.67 (-4.25 – 0.92)  **-2.84 (-5.42 – -0.26)*** |
| Waist circumference  Mean = 88.2 cm | I  II  III  IV | 0.44 (-0.07 – 0.95) 0.30 (-0.18 – 0.77) 0.24 (-0.19 – 0.66)  0.10 (-0.31 – 0.51) | 0.33 (-0.21 – 0.87) 0.35 (-0.19 – 0.89) 0.38 (-0.18 – 0.93) 0.38 (-0.19 – 0.95) | 1.54 (-1.36 – 4.44)  2.18 (-0.66 – 5.01)  1.75 (-1.16 – 4.66)  1.10 (-1.80 – 4.01) | -0.10 (-0.44 – 0.24)  -0.16 (-0.48 – 0.18)  -0.19 (-0.53 – 0.15)  -0.23 (-0.57 – 0.10) |
| BMI  Mean = 27.1 kg/m^2^ | I  II  III  IV | 0.94 (-0.34 – 2.21) 0.74 (-0.45 – 1.92) 0.39 (-0.65 – 1.43)  0.26 (-0.74 – 1.26) | 0.84 (-0.52 – 2.20)  0.85 (-0.50 – 2.20)  0.82 (-0.55 – 2.18)  0.81 (-0.59 – 2.22) | 4.24 (-3.04 – 11.51)  5.08 (-2.01 – 12.17)  4.54 (-2.61 – 11.69)  4.04 (-3.08 – 11.16) | -0.40 (-1.25 – 0.45)  -0.47 (-1.30 – 0.35)  -0.56 (-1.39 – 0.27)  -0.54 (-1.37 – 0.28) |

Linear regression coefficients (*β)* with 95% confidence interval (CI) indicates the difference in log cystatin C, serpin G1, CD14 or square root EV serpin F2 concentration per unit increase in adipose tissue parameter. Estimated differences are based on linear regression models. Model I: univariable model; Model II: adjustment for age and current smoking; Model III: model II with additional adjustment for eGFR and type 2 diabetes; Model IV: model III with additional adjustment for blood pressure lowering medication, lipid lowering medication and year of inclusion. Bold values indicate significance, *p<0.05.

**Table S2. Relation between plasma concentrations of hsCRP and EV-markers in patients with clinically manifest arterial disease.**

|  |  | Log cystatin C (pg/µg)  N = 1006  Mean = 2.24 | Log serpin G1 (pg/µg) N = 1007 Mean = 4.80 | Sqrt serpin F2 (pg/µg)  N = 1003 Mean = 6.09 | Log CD14 (pg/µg) N = 1009 Mean = 2.44 |
| --- | --- | --- | --- | --- | --- |
|  | Model | *β* (95%CI) | *β* (95%CI) | *β* (95%CI) | *β* (95%CI) |
| Log hsCRP N = 992 Mean log = 0.61 | I  II  III  IV | **9.33 (6.50 – 12.17)** 7.82 (5.04 – 10.59)** 6.18 (3.59 – 8.77)** 5.59 (3.00 – 8.18)**** | **10.00 (6.90 – 13.11)****  **8.83 (5.66 – 12.00)** 8.58 (5.41 – 11.75)** 7.96 (4.76 – 11.16)**** | **42.18 (27.49 – 56.86)****  **36.71 (21.82 – 51.60)** 37.05 (22.07 – 52.03)** 36.52 (21.49 – 51.55)**** | **7.72 (5.91 – 9.53)****  **6.34 (4.54 – 8.13)** 6.08 (4.28 – 7.87)** 5.72 (3.94 – 7.49)**** |
| Log adiponectin  N = 987 Mean log= 16.93 | I  II  III  IV | 2.12 (-0.88 – 5.11) 0.21 (-2.71 – 3.13) 0.65 (-2.08 – 3.38) 0.61 (-2.08 – 3.31) | -1.46 (-4.73 – 1.82)  -0.01 (-3.35 – 3.33)  -0.74 (-4.09 – 2.61)  0.58 (-3.93 – 2.76) | 1.73 (-13.50 – 16.96)  1.47 (-13.97 – 16.91)  1.90 (-13.75 – 17.54)  -0.108 (-16.00 – 15.38) | **2.25 (0.33 – 4.17)*** 1.72 (-0.17 – 3.61) **1.97 (0.07– 3.87)*** 1.49 (-0.36 – 3.35) |
| Log HOMA-IR  N = 464  Mean log= 0.87 | I  II  III  IV | **7.10 (1.12– 13.07)***  **6.85 (1.18 – 12.52)***  2.61 (-2.83 – 8.05) 2.31 (-3.20 – 7.82) | -3.51 (-10.22 – 3.21)  -4.56 (-11.27 – 2.15)  -5.50 (-12.45 – 1.45)  -5.40 (-12.45 – 1.64) | -10.97 (-43.67 – 21.73)  -13.21 (-45.85 – 19.44)  -21.60 (-55.52 – 12.33)  -20.23 (-54.81 – 14.35) | -2.78 (-7.18 – 1.61)  -3.15 (-7.45 – 1.16)  -4.73 (-9.18 – -0.28)  -3.71 (-8.16 – 0.74) |
| HDL-C  N= 1012  Median = 1.3  (1.0 – 1.5) | I  II  III  IV | **-10.45 (-18.07 – -2.82)***  **-18.49 (-26.06 – -10.91)****  **-13.10 (-20.27 – -5.94)****  **-11.26 (-18.39 – -4.13)*** | -6.80 (-15.18 – 1.58)  -2.10 (-10.87 – 6.67)  -2.55 (-11.43 – 6.33)  -0.34 (-9.26 – 8.59) | 20.83 (-18.34 – 60.01)  20.41 (-20.35 –61.17)  22.16 (-19.46 – 63.78)  28.96 (-12.54 – 70.46) | 4.43 (-0.50 – 9.37)  2.44 (-2.56 – 7.44)  4.09 (-0.98 – 9.16)  **5.04 (0.07 – 10.00)*** |

**Relation between metabolic parameters of adipose tissue (dys)function and EV-markers in patients with manifest cardiovascular disease.** Linear regression coefficients (*β)* with 95% CI indicates the difference in log EV-cystatin C, log EV-serpin G1, square root EV-serpin F2 or log EV-CD14 concentration per unit increase in log hsCRP, log adiponectin, log HOMA-IR or HDL-cholesterol. Model I: univariable model, Model II: adjusted for age, sex and current smoking, Model III: additional adjustment for eGFR, type 2 diabetes, Model IV: additional adjustment for blood pressure lowering medication, lipid lowering medication, platelet aggregation inhibitors and year of inclusion in SMART. Bold values indicate significance, *p<0.05; ** p<0.0001.

NB The relation between HOMA-IR and EV-cystatin C was only performed in patients without use of anti-hyperglycaemic drugs.

**Table S3. Relation between EV-markers and the metabolic syndrome in patients with clinically manifest arterial disease.**

|  | |  | Log cystatin C (pg/µg)  N = 1006  Mean = 2.24 | Log serpin G1 (pg/µg) N = 1007 Mean = 4.80 | Sqrt serpin F2 (pg/µg)  N = 1003 Mean = 6.09 | Log CD14 (pg/µg) N = 1009 Mean = 2.44 |
| --- | --- | --- | --- | --- | --- | --- |
|  | | Model | OR (95%CI) | OR (95%CI) | OR (95%CI) | OR (95%CI) |
| Metabolic Syndrome  No =492  Yes =517 | I  II  III | **1.55 (1.18 - 2.05)***  **1.61 (1.20 – 2.17)***  **1.55 (1.12 – 2.14)*** | 1.10 (0.87 – 1.41)  1.05 (0.82 – 1.35)  1.02 (0.79 – 1.30) | 1.02 (0.96 – 1.07)  1.01 (0.95 – 1.06)  0.99 (0.94 – 1.05) | 1.13 (0.75 – 1.72)  1.02 (0.66 – 1.58)  0.87 (0.56 – 1.37) |  |

Odds ratios with 95% CI indicate the odds for metabolic syndrome per increase in log cystatin C, serpin G1, CD14 or square root EV serpin F2 concentration.

Estimated differences are based on multivariable logistic regression models. Model I: univariable model; Model II: adjustment for age, sex and current smoking; Model III: model II with additional adjustment for hsCRP, eGFR and year of inclusion. Bold values indicate significance, *p<0.05.

**Table S4. Relation between EV-markers and risk of onset type 2 diabetes in patients with clinically manifest arterial disease.**

|  |  | cystatin C (pg/µg)  N = 528  Median 8.9 (7.0-11.0) | serpin G1 (pg/µg) N = 530 Median 113.0 (82.4-155.5) | serpin F2 (pg/µg)  N = 524 Median 31.1 (20.6-51.1) | CD14 (pg/µg) N = 530 Median 11.0 (9.1-13.2) |  |
| --- | --- | --- | --- | --- | --- | --- |
|  | Model | HR (95%CI) | HR (95%CI) | HR (95%CI) | HR (95%CI) |  |
| Type 2 diabetes  # of events 42 | | I  II  III | 1.03 (0.96- 1.10)  1.01 (0.94 – 1.09)  0.98 (0.90 – 1.07) | 1.00 (0.99 – 1.00)  1.00 (0.99 – 1.00)  0.99 (0.99 – 1.00) | 0.99 (0.98 – 1.00)  0.99 (0.97 – 1.00)  **0.99 (0.97 – 0.99)*** | **0.90 (0.81 – 1.00)***  **0.87 (0.78 – 0.97)***  **0.84 (0.75 – 0.94)*** |

Hazard ratios with 95%CI indicate the relative risk for incident type 2 diabetes per increase in EV-cystatin C, EV-serpin G1, EV-serpin F2 or EV-CD14 concentration during 6.5 years (interquartile range 5.8–7.1 years) follow-up, based on multivariable Cox proportional hazard regression. Model I: univariable model, Model II: adjusted for age, sex, current smoking, Model II: additional adjustment for hsCRP, eGFR, year of inclusion in SMART, metabolic syndrome and HOMA-IR. Bold values indicate significance, *p<0.05.
